# Inverse relationship between Alzheimer’s disease and cancer, and other factors contributing to Alzheimer’s disease: a systematic review

**DOI:** 10.1186/s12883-016-0765-2

**Published:** 2016-11-22

**Authors:** Ovais Shafi

**Affiliations:** Sindh Medical College, Dow University of Health Sciences, Karachi, Pakistan

**Keywords:** Alzheimer’s disease, Cancer, Neuro-degeneration, Aging, Senile plaques, Neurofibrillary tangles

## Abstract

**Background:**

The AD etiology is yet not properly known. Interactions among environmental factors, multiple susceptibility genes and aging, contribute to AD. This study investigates the factors that play role in causing AD and how changes in cellular pathways contribute to AD.

**Methods:**

PUBMED database, MEDLINE database and Google Scholar were searched with no date restrictions for published articles involving cellular pathways with roles in cancers, cell survival, growth, proliferation, development, aging, and also contributing to Alzheimer’s disease. This research explores inverse relationship between AD and cancer, also investigates other factors behind AD using several already published research literature to find the etiology of AD.

**Results:**

Cancer and Alzheimer’s disease have inverse relationship in many aspects such as P53, estrogen, neurotrophins and growth factors, growth and proliferation, cAMP, EGFR, Bcl-2, apoptosis pathways, IGF-1, HSV, TDP-43, APOE variants, notch signals and presenilins, NCAM, TNF alpha, PI3K/AKT/MTOR pathway, telomerase, ROS, ACE levels. AD occurs when brain neurons have weakened growth, cell survival responses, maintenance mechanisms, weakened anti-stress responses such as Vimentin, Carbonic anhydrases, HSPs, SAPK. In cancer, these responses are upregulated and maintained. Evolutionarily conserved responses and maintenance mechanisms such as FOXO are impaired in AD.

Countermeasures or compensatory mechanisms by AD affected neurons such as Tau, Beta Amyloid, S100, are last attempts for survival which may be protective for certain time, or can speed up AD in Alzheimer’s microenvironment via C-ABL activation, GSK3, neuro-inflammation.

**Conclusions:**

Alzheimer’s disease and Cancer have inverse relationship; many factors that are upregulated in any cancer to sustain growth and survival are downregulated in Alzheimer’s disease contributing to neuro-degeneration. When aged neurons or genetically susceptible neurons have weakened growth, cell survival and anti-stress responses, age related gene expression changes, altered regulation of cell death and maintenance mechanisms, they contribute to Alzheimer’s disease. Countermeasures by AD neurons such as Beta Amyloid Plaques, NFTs, S100, are last attempts for survival and this provides neuroprotection for certain time and ultimately may become pathological and speed up AD. This study may contribute in developing new potential diagnostic tests, interventions and treatments.

**Electronic supplementary material:**

The online version of this article (doi:10.1186/s12883-016-0765-2) contains supplementary material, which is available to authorized users.

## Background

The etiology of Alzheimer’s disease is yet not properly known. Alzheimer’s disease is a neurodegenerative disease and cancers are over-proliferative. Understanding the relationship between cancer and AD, in terms of cellular pathways and molecular mechanisms will help to understand better the pathogenesis and factors behind Alzheimer’s disease. This study also investigates how age related changes in growth, cell survival responses, maintenance mechanisms, anti-stress responses, contribute to development and progression of Alzheimer’s disease.

In the light of scientific literature, it points that changes and appearance of tau, beta amyloid, S100, C-ABL, GSK3, neuro-inflammation in neurotoxic environment of AD may just be the countermeasures by AD affected neurons as last attempts for survival. This research paper also points to possible mechanisms and pathways that may be playing role or contributing to Alzheimer’s disease pathogenesis. But these possible mechanisms and pathways need to be further investigated.

It is already known that in severe cases of AD amyloid plaques may be undetectable, but may be present in brains of those lacking dementia, and NFTs have been found to be linked to longevity of neuron. Other factors may be causing bulk of neurodegeneration in this disease, not the NFTs. There is exponential rise with age in the occurrence of both AD and cancer. Epidemiological studies have shown a reciprocal association between Alzheimer’s disease and cancer; elderly with AD dementia have a decreased risk of cancer and vice versa [1, 2].

It is already known that among Huntington disease patients; there is lower occurrence of cancer [3]. This makes it more essential to investigate the inverse relationship between Cancer and AD.

This study may also possibly contribute to the development of new diagnostic tools and new treatments for Alzheimer’s disease. The circumstances which lead to the development of AD remain unclear yet and this study also tries to investigate them. It also highlights the cellular and molecular pathways that are involved in AD and Cancer, and also simultaneously pointing to their relationships in both the disease processes. It also suggests some hypothesis in the light of scientific literature and also highlights the changes occurring at cellular and molecular level that contribute to development of AD.

## Methods

PUBMED database, MEDLINE database, Google Scholar and other online Journals such as BMC, Neurology today and others were searched with no date restrictions for published articles using keywords P53, estrogen, neurotrophins, growth factors, growth and proliferation, cAMP, EGFR, Bcl-2, apoptosis pathways, IGF-1, HSV, TDP-43, APOE, notch signaling, presenilins, NCAM, TNF alpha, PI3K/AKT/MTOR pathway, telomerase, ROS, ACE levels, vimentin, carbonic anhydrases, HSPs, SAPK, Carnosine, neuro-protection, antineoplastic, FOXO, Tau, Beta Amyloid, S100, C-ABL activation, GSK3, neuro-inflammation. All these keywords were searched with terms such as cancer, Alzheimer’s disease, and those mentioned in eligibility criteria. For example, “IGF-1 and Alzheimer’s disease”, “IGF-1 and Cancer”. Some additional articles of interest were selected from reference lists of included articles.

Only those articles were eligible to be included which involved cellular pathways having roles in cancers, development, aging, cell survival, growth and proliferation, and also contribute to Alzheimer’s disease. Screening of the literature was also done on the same basis. Literature search began in January 2013 and ended in January 2016. The prime focus of the literature search was to screen the literature on the basis of eligibility criteria. Publications only in English were used and there was not any limitation on date of publication. Data extraction was based on expression of factors common in cellular pathways having roles in cancers, cell survival, growth, proliferation, development and aging. And also contribute to Alzheimer’s disease. If many separate studies were present with similar conclusions, then only those were selected to be included which were most relevant to the objectives of the study and research question. No unpublished study was used or included. This manuscript adheres to PRISMA guidelines. This research study finds evidence from already published research literature to find factors and changes that lead to the development of Alzheimer’s disease and finds the inverse relationship between Cancer and Alzheimer’s disease.

## Results

A total of 1824 articles were identified using database searching, 1590 were recorded after duplicates removal. One thousand three hundred sixty-six were excluded after screening of title/abstract, 119 were finally excluded (because when many separate articles were present with similar conclusions, then only those were selected to be included which were most relevant to the objectives of the study and research question), 4 were excluded during data extraction. Finally 101 articles were included (because they involved cellular pathways having roles in cancers, development, aging, cell survival, growth, proliferation and also contributing to Alzheimer’s disease).

### Inverse relationship between cancer and Alzheimer’s disease

There is inverse relationship between Cancer and Alzheimer’s disease in aspects such as P53 is upregulated in Alzheimer’s disease and down-regulated in Cancer, estrogen is neuro-protective but increases the risk of cancers, neurotrophins and growth factors are neuroprotective but are also involved in tumor growth progression, age related decline in proliferation of new cells contribute to AD development while pathways and mechanisms that contribute to growth and proliferation delays AD, *cAMP* provides survival signal for neurons and is also involved in tumor progression, EGFR is overexpressed in cancer but EGFR is not found in Alzheimer’s plaques, Bcl-2 downregulated in Alzheimer’s disease but is overexpressed in cancer, apoptosis pathways are upregulated in Alzheimer’s disease but downregulated in cancer, IGF-1 is decreased in Alzheimer’s disease but increased in cancer, dysfunctional proliferation of neurons occurs in Alzheimer’s but in cancer there is over-proliferation of cells, HSV is oncolytic but contributes to Alzheimer’s disease development, TDP-43 role in Alzheimer’s disease and cancer and its relation to IGF signifies the inverse relationship between cancer and AD, Alzheimer’s risk decreases from apoE4 to E3 to E2 but growth and survival improves respectively, pathophysiologic notch signals potentially contribute to cancer but presenilins are also involved in notch signalling and they mutate in familial early-onset AD, neural cell adhesion molecule decrease in AD but stain positive in neoplasia, Tumor Necrosis Factor-α has anti-cancer properties and its overexpression causes neurotoxic environment but secondary signal is necessary for the induction of neuronal death, PI3K/AKT/MTOR pathway is neuroprotective but in many cancers this pathway is overactive, telomerase in cancer cells prevents senescence related death and AD is associated with accelerated neuronal death, ROS when excessive slows cancer proliferation and ROS are increased in Alzheimer’s disease, ACE levels are decreased in Cancer but are elevated in Alzheimer’s disease.

Epidemiological studies have also pointed towards the inverse relationship between Alzheimer’s disease and Cancer [1, 2]. All those factors that contribute to growth and proliferation are increased in cancers but decreased in Alzheimer’s disease. This simply does not mean that every cellular or molecular pathway should have inverse relationship; there are so many pathways that are common and even operate similarly in many cell types, and are not altered by the disease processes.

#### Apoptosis pathways including P53 are upregulated in Alzheimer’s disease and down-regulated in cancer

P53 downregulation is the foundation of most tumors. Inactivation of *TP53* confers a predisposition to cancer, while Alzheimer’s disease (AD) leads to apoptosis induction by the p53 pathway. Massive neuronal death represents important neuropathological hallmarks of AD. P53 can initiate apoptosis if DNA damage proves to be irreparable. P53 activation leads to cell cycle arrest via p21 and same p21 stops cells from dividing after being exposed to damaging agents. Upregulation of p21 results in aging and cell cycle arrest [4]. It is already known that deletion of p53/p21 in mouse models improves aging phenotypes and extends the life span but increases the risk of cancer.

Amyloid Precursor Protein (APP) mediates partly p53 expression, binding of Aβ especially to promoter of p53 increases transcription of p53, and tau phosphorylation is indirectly stimulated by p53 [5]. Complete deletion of Tp53 occurs in Li-Fraumeni syndrome, lifetime cancer risk increases to 100% by age 70 years in these patients [6]. This signifies the inverse relationship between AD and Cancer, as upregulation of P53 is present in AD and its downregulation or deletion is involved in cancers.

In human neuroblastoma cells, Aβ42 has been found to lower the expression of the X-linked inhibitor of apoptosis (XIAP), and overexpression of XIAP reduces the vulnerability to oxidative stress caused by Aβ_42_ [7]. It is important to note that apoptosis is upregulated in Alzheimer’s disease and down regulated in Cancer cells.

#### Estrogen is neuro-protective but increases the risk of cancers; this signifies the inverse relationship between AD and cancer

Estrogen is both neurotrophic and neuroprotective. It even protects isolated neurons in vitro from hypoglycemic and ischemic injuries, oxidative stress, and from damage by Aβ42; which is implicated in the AD pathogenesis. Nerve growth factors promote neuronal viability, repair and growth of damaged neurons, and dendritic branching. Estrogen also increases their production.

An imbalance occurs in AD between neuronal injury and repair. The role of Estrogen in reducing the risk of AD is well established [8]. It is already known estrogen increases the risk of Cancers i.e. ovarian, endometrial, breast [9].

#### Neurotrophins and growth factors are neuroprotective and are also involved in tumor growth progression

NGF is also involved in regulating tumour growth and progression of cancers including lung, medullar thyroid carcinoma, prostatic, breast and pancreatic carcinomas. Interactions of neurotrophic factors and glutamate are also involved in regulating developmental and adult neuroplasticity. For example, production of brain-derived neurotrophic factor (BDNF) is stimulated by glutamate, and BDNF modifies glutamate sensitivity of neurons, neuronal plasticity, and Ca^2+^ homeostasis. Neurotrophic factors also change the expression of glutamate receptor subunits and Ca^2+^-regulating proteins, and also induce the production of anti-apoptotic Bcl-2 family members, antioxidant enzymes and energy-regulating proteins. Oxidative and metabolic stress activate glutamate receptors in excess, may contribute to neuro-degeneration and dysfunction in stroke psychiatric disorders and Alzheimer’s disease [10].

Enhancement of environmental factors such as exercise, neurotrophic factor signaling, antidepressants, dietary energy restriction have the potential to optimize glutamatergic signaling and protect against neurological disorders. Physiologically BDNF is involved in neuronal growth and survival. In areas vital to learning, memory, and higher thinking such as hippocampus, cerebral cortex, and basal forebrain; BDNF actively plays its role. It is significant for long term memory and neurogenesis [11, 12].

Current knowledge suggests strongly that epidermal growth factor (EGF) and the neurotrophins – nerve growth factor (NGF), brain derived nerve growth factor (BDNF), neurotrophin-3 (NT-3) and neurotrophin 4/5 (NT-4/5), as well as their tyrosine kinase receptors – epidermal growth factor receptor (EGFR) and members of the trk family (trk A, trk b and trk c) play a significant role in stromal–epithelial interactions during Prostate Cancer pathogenesis [13].

Expression of BDNF also occurs in cancers such as colon cancer, and alters the behavior of cancer cells at cellular level. It also reduces the apoptosis of colon cancer cells [14]. There is significant role of inflammation in AD pathogenesis. In AD, there is down-regulation of BDNF and other neurotrophins. There are lower levels of BDNF in the brain tissues of people with Alzheimer’s disease [15], and it has already been known that BDNF is anti-apoptotic, also plays role in progression of cancer.

#### Age related decline in proliferation of new cells contribute to AD development, while pathways and mechanisms that contribute to growth and proliferation delays AD

Exercise enhances the size of hippocampus, and improves memory function. BDNF plays a significant role in this process [16]. Physical activity reduces risk of Alzheimer’s disease. There are significant gene expression changes in exercising and non-exercising muscles, because of exercise [17]. Exercise makes changes in genome at epigenetic level — particularly in genes linked to fat storage and a high risk of developing diabetes or obesity [18]. In subgranular zone of hippocampal dentate gyrus, generation of new neurons continues throughout lifetime. Neurogenesis lowers with aging, and this decrease also contributes to mild cognitive impairment. Neurodegenerative diseases are associated with decreased neurogenesis, and increasing hippocampal neurogenesis with methods like environment enrichment, exercise, diet restriction, slows AD progression.

Exercise increases memory function and plasticity of hippocampus. With increasing age, hippocampal neurogenesis is decreased and exercise attenuates age-related reduction in hippocampal generation of neurons [19].

#### TGF beta: Anti- proliferative physiologically—increased levels in AD but in cancers its normal anti-proliferative functioning is disrupted and is redirected to contribute to tumorigenesis

In normal cells, TGF-β halts cell cycle at the G1 stage to stop proliferation and induces cell to differentiate or go towards apoptosis. In many cancer cells, TGF-β is mutated and its normal functioning is impaired. It contributes to make cancer more aggressive via causing immunosuppression and angiogenesis, also influences surrounding stromal cells, immune cells, endothelial and smooth-muscle cells [20]. TGF-β also converts effector T-cells into suppressor T-cells, which halt the inflammatory reaction. Increased levels of TGF-β are found in the cerebrospinal fluid and blood of AD patients as compared to control subjects [21], suggesting a possible role in the neurodegenerative processes leading to Alzheimer’s disease development. The role TGF beta plays in AD and cancer signifies the inverse relationship between these diseases.

#### *cAMP* provides survival signal for neurons and also involved in tumor progression

Cyclic AMP (cAMP) is such a powerful survival signal for neurons, that even the survival of in vitro neurons is greatly enhanced by increased intracellular cAMP levels. cAMP enhances neuronal survival, but survival is significantly enhanced when cAMP is in combination of multiple peptide trophic factors [22]. Cyclic AMP elevation sufficiently enhances the neuronal survival even in vitro. *M*any peptide trophic factors, including brain-derived neurotrophic factor (BDNF), ciliary neurotrophic factor (CNTF), fibroblast growth factor (FGF), glial-derived neurotrophic factor (GDNF), and hepatocyte growth factor (HGF), promote the short-term survival of highly purified embryonic spinal motor neurons (SMNs) in culture [23]. Cyclic AMP is already known to have role in tumor progression. Aberrant activation of cAMP-controlled genes and deregulation of cAMP pathways is linked to the growth of some cancers [24].

#### EGFR is involved in growth, proliferation and survival of cells and it is overexpressed in cancer but becomes deficient in AD. This also signifies the inverse relationship between Alzheimer’s disease and cancer

Several cancers, including glioblastoma multiforme, lung cancer and anal cancers, are associated with overexpression of epidermal growth factor receptor (EGFR). In cancers of epithelial origin, EGFR overexpression plays important role in their pathogenesis [25]. But EGFR is absent in central core of AD neuritic plaques [26]. EGF; a mitogen for neural stem and progenitor cells, also plays role in hippocampal neurogenesis and cognitive function’s improvement [27].

#### Bcl-2 downregulated in Alzheimer’s disease but overexpressed in cancer

BCL-2 overexpression occurs in many cancer types and is associated with chemo-resistance and radio-resistance. Increased levels of Bcl – 2 lower the propensity of cancer cells to initiate apoptosis. Expression of Bcl-2 and other oncogenes is increased in cancer; this contributes to cancer cell survival. But in AD, there is down-regulation of Bcl-2 [28]. Bcl-2 overexpression has already been shown to provide protection against beta amyloid induced cell death, and this effect is possibly related to decrease in beta amyloid induced activation of p38 MAPK and NF-κB [29].

#### IGF-1 is decreased in Alzheimer’s disease but increased in cancer, signifying the inverse relationship between AD and cancer

Lack of Insulin or Insulin like Growth factors potentiate the AD. High IGF levels are present in cancers. Congenital deficiencies in IGF-1 protects against the development of cancer [30]. In diabetic mouse models, aging has been found to cause impairment of the respiratory chain and to lower efficiency of oxidative phosphorylation and also the mitochondrial capacity to accumulate Ca^2+^. Beta amyloid’s presence accelerates the age related mitochondrial effects. When in the presence of Aβ40, brain mitochondria were isolated from diabetic rats; increased levels of H_2_O_2_ were observed. But insulin and coenzyme Q10 (CoQ10) treatments prevented these pathological effects [31].

IGF – 1 plays significant role in normal physiological mechanisms and also in disease states, including cancer. IGF axis inhibits cell death and promotes cell proliferation, it also is involved in neural development including neurogenesis, myelination, synaptogenesis, and dendritic branching and neuroprotection after neuronal damage. Higher IQ has also been associated with increased serum levels of IGF-I in children. IGF-2 is also significant for function and development of organs such as the liver, kidney and brain. Risk of AD dementia is also associated with lower serum levels of IGF-1, and higher levels of IGF – 1 are associated with greater brain volumes and lower risk of dementia and stroke. Higher IGF-1 levels may protect against neurodegeneration [32].

It is already known that diabetes especially type 2, is associated with increased risk of developing AD and other dementias [33]. This signifies the inverse relationship between Alzheimer’s disease and cancer, as IGF contributes to cell proliferation and halts cell death. Its levels are increased in cancer and plays role in carcinogenesis but IGF levels are decreased in Alzheimer’s disease and there is accelerated neuronal death. Aging is a major risk factor for AD as expression of COX subunits II and IV is decreased during aging and such age-related changes are more marked in AD [34].

Leigh syndrome includes the loss of mental function but not with AD specific symptoms, though there is cytochrome c oxidase deficiency. This potentially suggests that COX reduction is a consequence of the disease not the key causative factor.

#### Dysfunctional proliferation of neurons occurs in Alzheimer’s, and Cancer is over-proliferative disease

In Alzheimer’s disease, proliferation of neurons becomes dysfunctional, despite some neurogenesis there is overall great neuronal loss. But in cancer, the cells gain the capacity to over-proliferate and they also regulate the microenvironment if favor of their survival. This signifies the inverse relationship between Alzheimer’s disease and cancer.

Morphogens play significant role in embryonic development of nervous system, including Wnts, Notch, Shh, and BMPs. They are conserved and continue to play role as niche signals for adult neural stem cells to regulate maintenance, activation, and fate choice of NSCs. Neurogenesis is also regulated by various neurotrophins, cytokines, hormones, neurotransmitter systems and growth factors. Transcription factors, miRNAs, epigenetic factors and cell-cycle regulators provide adult NSCs with the potential to differentiate, proliferate and survive as newborn neurons. Newborn neurons differ substantially from their older, neighboring counterparts in terms of electrophysiological properties. They show increased plasticity. ApoE, PS1, APP and its metabolites, can modulate adult hippocampal neurogenesis, these molecular players known to contribute to AD pathogenesis. Risk of AD may be exacerbated by Dysfunctional neurogenesis resulting from early subtle disease manifestations, whereas increased neurogenesis represent an endogenous brain repair mechanism and possibly be compensatory response. NFTs, beta amyloid plaques and massive neuronal death are significant neuropathological hallmarks of AD. These pathologies deeply harm hippocampus among other specific vulnerable areas in brain. Adult hippocampal neurogenesis play role in improvement of pattern separation and spatial memory. Aging decreases neurogenesis, and this in turn contributes to AD.

Neurogenesis has the potential to delay or halt AD-linked cognitive decline. But it is altered at the very early stage of AD progression, even before appearance of neuronal loss, amyloid deposition and inflammation. Dysfunctional neurogenesis is a significant part of AD pathology. Endogenous neurogenesis stimulation via environmental stimuli, physical activity, trophic factors, cytokines, and drugs may promote the recovery and regeneration process [35].

AD-related inflammation may explain the increase in hippocampal neurogenesis. Inflammatory factors changes patterns of proliferation or survival of new cells, and in turn may affect neurogenesis. Transforming growth factor beta 1 (TGFβ-1), a strong inflammatory factor present in AD enhances neurogenesis [36]. Role of immune system in neurogenesis is complex and depends on multiple factors [37].

Role of neurotransmitters as growth regulatory signals are well established, they have neuroprotective effects against dementia. As it’s already known when many AD patients died they had lower than normal levels of Acetylcholine. Estrogen’s neuroprotective role against AD is also well established.

#### HSV is oncolytic and also contributes to Alzheimer’s disease development, this signifies the inverse relationship between cancer and AD

HSV 1, mediates selective oncolysis of gliomas. Genes related to mammalian DNA damage and growth arrest; *GADD3,* are very homologous to C-terminal 70-amino acids of HSV 1. Cellular proteins interact to halt the protein synthesis by double-stranded RNA-activated protein kinase [38]. Within Alzheimer’s disease amyloid plaques, HSV 1 DNA has already been found [39]. When cultured neurons and glial cells are infected with HSV 1, it leads to elevated levels of beta-amyloid 1–40 and 1–42 within the cells, but there is decrease in amyloid precursor protein (APP) levels. Even after HSV 1 infection, beta amyloid plaques appear in mouse brain [40].

Role of HSV 1 is oncolytic and it also has the capacity to contribute to Alzheimer’s disease pathogenesis as the pathways that work against cell proliferation and survival, are involved in AD development. This signifies the inverse relationship between Alzheimer’s disease and cancer.

#### TDP-43 role in Alzheimer’s disease and cancer and its relation to IGF, signifying the inverse relationship between cancer and AD

Abnormal TDP - 43 causes genomic instability and increased apoptosis, and this also plays role in AD development. Deficiency of IGF potentiates the damage by this pathway. Normally TDP 43 regulates CDK 6. CDK 6 is upregulated in Cancer and down-regulated in AD. Abnormal TDP - 43 increases in AD and loss of TDP-43 contributes to cancer via upregulation of CDK6.

When TDP-43 becomes abnormal, the severity of clinical deficits increases and there is greater atrophy in brain, particularly in hippocampal region [41]. Levels of Cdk6 increase 10-fol when TDP-43 is absent. Physiologically, Cdk6 plays role in determining differentiation of several cell types. But elevated levels of Cdk6 expression are associated with several tumors [42]. There is strong association of abnormal TDP- 43 with AD related cognitive impairment [43].

Insulin and IGF-1 signaling is also considered to have roles in contributing to biological aging in several organisms, and it is already known that misfolding of proteins contributes to age- related degeneration of neurons associated with TDP-43, and a significant decrease/deficiency in IGF-1 and insulin levels has the potential to lower the neurotoxicity of abnormal TDP-43 aggregation [44]. Beta amyloid related oxidative stress and impairment in mitochondrial OXPHOS efficiency can potentially be prevented by IGF and coenzyme Q10 related treatments [31]. It is already known that IGF levels are increased in cancer and decreased in AD.

It is the biological aging mechanisms that are ultimately contributing to the pathogenesis of AD and cancer, and even the risk of both also increases significantly with increasing age.

#### Alzheimer’s risk decreases from apoE4 to E3 to E2 but growth and survival improves from E4 to E3 to E2

It is already known APOE influences AD risk [45], but the underlying mechanisms are not yet determined. The pathways and mechanisms that play roles in growth, survival, and oncogenesis, when downregulated or not functioning properly; they contribute to development of Alzheimer’s disease. This finding has been elaborated in terms of ApoE variants.

It is important to note that APOE4 has highest risk for AD, and apoE4 also lowers neurite outgrowth. APOE3 is very common but poses less risk compared to E4, and E3 also inhibits the proliferation of tumor cells among other cell types. But apoE3 increases neurite outgrowth mostly, but not always.

E2: This variant is rare and least dangerous. In colon cancer this apoE contributes to cell growth. E2 and E4 variants have opposite actions, and this further supports the role of APOE in AD pathogenesis. Alzheimer’s risk decreases from apoE4 to E3 to E2 but growth and survival improves respectively. Genotype APOE2 and E3 are associated with lowest risk of AD [46].

APOE plays its role in repair mechanisms after tissue injury. It also modulates cell differentiation, growth and is also involved in immune regulation. APOE is involved in neuroprotection, neurite outgrowth, neural development and regeneration. It plays role in remodeling after neuronal injury, to restore neuronal function [47]. But APOE4 is unable to do these functions properly.

Expression of APOE has already repeatedly been detected in serous carcinomas of ovary. ApoE-specific siRNA when used to halt ApoE expression, it arrested the cell cycle and led to apoptosis in an ovarian cancer cell line. APOE plays role in proliferation and survival of apoE-expressing ovarian cancer cells [48], and APOE is also associated with AD; a neurodegenerative disease.

APOE also contributes to maintenance and proliferation of some adult neural stem cells [49]. APOE4 variant is associated with highly increased risk of AD. APOE3 is more common and has lesser AD risk. E2 variant is associated with the least AD risk. It is important to note that inheritance of APOE4 increases the risk of AD development, only the risk but not the disease itself, as not everyone with this allele develops AD. This most likely shows APOE4 itself is not an exact causative factor but it may be an associated or contributing factor. Malignant and normal human intestine, both express APOE. Expression of APOE also impacts cultured human colonic adenocarcinoma cells.

Macrophage-derived Apo-E has the capability to regulate integrity of epithelial cells and this way also contributes to growth of cells [50]. Apolipoprotein E3: It is involved in inhibiting the proliferation of tumor cells and endothelial cells among other cell types in a dose and time dependent manner. E3 can strongly inhibit the proliferation of many cell types. Metastasis, angiogenesis and tumor cell growth may effectively be modulated by APOE3 [51].

Role of APOE4 in pathogenesis of AD, MS and in reducing hippocampal volume is already well known. We already know that all these diseases are associated with destruction of neurons. Poor prognosis in patients of traumatic brain injury is also related to APOE4 genotype.

It is already known that brain needs regeneration and repair to counter TBI, and the presence of APOE4 linked to poor outcome, this also points to its anti-proliferative effects. APOE4 in female carriers has been shown to contribute to accelerated telomere shortening and biological aging, hormone replacement therapy halts this accelerated aging [52].

While in cancer there is increased activity of Telomerase causing elongation of telomeres in cancer cells. This also signifies the link between hormones and AD as those APOE4 carriers who continued to use hormone replacement therapy; did not show signs of accelerated aging. Reduced levels of androgens also potentially increase the risk of AD.

APOE4 contributes to reduced neurite outgrowth: APOE isoforms regulate neuronal ability to regulate remodel, and APOE4 allele impairs with ability in AD patients. E4 variant even dominates its effects when present with E3. It is important to note that E3 variant is associated with increased growth of neurite [53].

The APOE4 and APOE2 variants have opposite actions, and their roles in growth and survival also signify the inverse relationship between cancer and AD.

#### Pathophysiologic notch signals potentially contribute to cancer but presenilins are also involved in notch signalling and mutated PS is involved in familial early-onset AD

Presenilins are also implicated in the processing of notch, an important developmental protein. Familial early-onset AD is associated with mutated Presenilin 1 (*PS1*) and Presenilin 2 (*PS2*) genes. Normal PS 1 and PS 2 are also involved in intramembranous cleavage of Notch. Notch signaling pathway is very significant for multiple cell differentiation processes, gene regulation and cell to cell communication. Its role in neuronal function and development is already well established. When this pathway is inhibited, this results in anti-proliferative effects. Notch signaling is also involved in CNS plasticity. Mutations in notch1 have been shown to result in spatial learning and memory deficits. Such similar also appear in experiments involving PS 1 and PS 2, specifically when Presenilins are deleted at 3 weeks post-birth, this causes dysfunction and degeneration of neurons gradually, also causes deficits in learning and memory. Notch signaling plays a significant role in developmental pathways and also play roles in cancers [54].

Cytokines, hormones, and neurotransmitters, morphogens, proteoglycans, and growth factors are involved in influencing the proliferation and survival of neural stem cells, and they are called Neural Stem Cell Survival factors. Notch signaling diversely effects cellular proliferation, survival, and differentiation, it also plays an important role in normal developmental processes of many cell types. When this signaling becomes pathophysiologic, it then also contributes to malignant transformations [55, 56].

This signifies the inverse relationship between cancer and AD, as mutant PS is involved in AD and normal PS itself is involved in processing of Notch, the pathophysiology of which is implicated in cancer.

#### Neural cell adhesion molecule (NCAM), also called CD56, decrease in AD but stain positive in neoplasia

APOE4 genotype is associated with low NCAM levels. AD is also associated with low levels of BDNF in temporal and frontal brain regions [57]. NCAM is also involved in inducing neurite outgrowth via FGFR and also acts on other signaling pathways.

CD56 (NCAM) immunohistochemistry is also used by pathologists to identify specific tumors such as pheochromocytoma, paraganglioma, T cell/ natural killer lymphoma, myeloid leukemia, myeloma, neuroendocrine tumors, Wilms’ tumor, neuroblastoma, pancreatic acinar cell carcinoma, Ewing’s Sarcoma and small cell carcinoma of lung.

It is important to note that normal cells also having positive staining for CD56 include neuroendocrine tissues, cerebellum, brain, NK cells and activated T cells. Beta-amyloid even harms NCAM2 and there are decreased levels of NCAM2 in hippocampus of AD patients [58].

#### Tumor Necrosis Factor-α overexpression causes neurotoxic environment but neuronal death occurs when secondary signal is also present

TNF alpha negatively impacts neurotrophins and promotes apoptosis. The inverse relationship between Cancer and Alzheimer’s disease in terms of neurotrophins and apoptosis has already been mentioned in this paper. Neurotoxic microenvironment and secondary signals play significant roles in causing TNF alpha to become harmful for brain cells. Dentate gyrus has been shown to have accelerated development when expression of TNF-α is absent, and this has been correlated to increased levels of nerve growth factor. Though, when TNF-α expression was absent, this absence impacted the branching of dendrites in hippocampal region. TNF-α over-expression has been shown to; decrease branching of dendrites, antagonize the production of NGF and lower neurotrophin levels in hippocampal region. TNF-α knock-out mice have also shown signs of increased memory function in hippocampus. TNF-α overexpression is associated with impairment in memory and learning function in hippocampus.

TNF-α signaling also has the capacity to negatively impact neuronal function. Pro-inflammatory cytokines lower long-term potentiation, and in turn also impair memory and learning. In AD, when amyloid plaques begin to accumulate, memory and learning deficits start becoming clear, and it is also the same time when TNF-α involvement occurs.

It is important to note that when TNF alpha levels are experimentally increased or it is overexpressed, it produces neurotoxic environment. TNF-α exposure has been suggested to be involved in beta amyloid plaques production.

TNF- alpha inhibition has also shown to improve cognitive status of AD patients [59]. TNF alpha and Apoptosis response: Many studies have also reported that besides TNF- alpha, an appropriate cellular environment or a secondary signal should also be present to initiate apoptosis, and when TNF – alpha is present alone, it has the potential to prevent induction of cell death after cellular insults. Degenerating environments and oxidative stress likely sensitize brain cells to inflammation driven apoptosis. Absence of TNF – alpha expression has been to enhance neuronal death when cerebral ischemia was induced in mice models. TNF alpha when expressed alone, it provides protection against neurotoxicity and even against beta amyloid plaques. Impact of TNF-alpha on neuronal viability is dependent on the presence or absence of secondary signals, which may arise from endogenous stimuli or exogenous situational stimuli [59].

#### PI3K/AKT/MTOR pathway is neuroprotective and in many cancers this pathway is overactive, signifying the inverse relationship between Alzheimer’s disease and cancer

The process of phosphorylation of specific proteins by activated AKT is involved in angiogenesis, cell cycling, metabolism, and cell survival, and it provides neuroprotection. AKT significantly regulates cell death and survival. The evolutionary conserved mTOR is a kinase that is involved in controlling the translation process of several mRNA transcripts having involvement in proliferation and growth of cell. Activated PI3K induces the activation of AKT [60]*.* PI3K/AKT/MTOR pathway is an intracellular signaling pathway that is neuroprotective. This pathway decreases apoptosis and promotes proliferation, there is over activation of this pathway in many cancers. The role of this pathway in cancer appears to be crucial [61].

#### Telomerase in cancer cells prevents senescence related death and AD is associated with accelerated neuronal death

In cancer cells, the function of telomeres is overcome by overexpression of an enzyme known as telomerase. Telomerase replaces the portion of the telomeres that is lost with each cell division, thereby avoiding senescence and permitting an infinite number of cell doublings. Telomerase is a target for antineoplastic drug development. It’s important to note that cancer cells have the ability to counter aging related death of their cells, by overexpression of an enzyme known as telomerase [62].

Alzheimer’s disease is associated with accelerated neuronal death. Shortening of telomere increases the instability of genome and formation of tumors, this has been shown in mouse models. This signifies the role of telomere length and activity of telomerase in the maintenance of genomic integrity. Telomere dysfunction contributes to genome instability in human cancer. But neoplastic cells have increased telomerase activity. There is huge overlap between genes changing expression with AD and those with age, direction of these changes is most often same. Aging related changes in gene expression may actually be increasing the risk of AD development [63].

In AD, epigenetic modifications impact both chromatin and DNA at molecular level, including cofactors and transcription factors. Transcription in neurons is regulated by amyloid intracellular domain (AICD) and Amyloid precursor protein (APP).

Role of chromatin remodeling and epigenetics in development of neurodegenerative diseases has emerged to be very significant. Changes in machinery of epigenome alter methylation of DNA and acetylation of histone, and this ultimately alters the gene transcription of APP and other genes involved in AD [64, 65].

Spontaneous tumors have deep evolutionary roots. Multi-cellular life and cancer, history of both is very old. Even in a primitive animal hydra, a primordial cancer has been discovered. There are great alterations in transcriptome of hydra tumors. These alterations mimic expression changes in vertebrate cancer [66].

Telomerase in cancer cells prevents senescence related death and AD is associated with increased neuronal death, this signifies the inverse relationship between Alzheimer’s disease and cancer. It may be hypothesized here that both disease processes are somewhat interlinked, as the major prevalence of both cancer and AD increases in people older than 55. In progeria, aging occurs several times faster than normal. If alone aging would have been the only factor predisposing to cancer and AD, then at least any of them would have been affecting progeria kids, but progeria patients show no neurodegeneration or cancer predisposition. But this needs to be further investigated.

#### Reactive oxygen species when excessive slows cancer proliferation and ROS are increased in Alzheimer’s disease

Dysfunction of mitochondria and oxidative stress contributes to the pathogenesis of Alzheimer’s disease. With increasing age, ROS increases in brain and this accumulation of ROS accelerates with AD. All this might be due to aging and AD related neuronal damage [67]. Once a cell has become cancerous then excessive stress by ROS slows proliferation and threatens survival [68]^.^

#### ACE levels are decreased in cancer but are elevated in Alzheimer’s disease, signifying the inverse relationship to both AD and cancer

Surface of many cell types, such as macrophages, dendritic, endothelial, neuro-epithelial, epithelial cells express constitutively ACE (CD 143) as a membrane bound protein. Most of the cancer patients tested presented with the ACE mean value for all patients being 70% of the standard value [69]. In AD, activity and levels of ACE are generally increased in the cerebral cortex [70]. The role of ACE (CD 143) is not yet fully established in Alzheimer’s disease.

#### Another neuro-degenerative disease having reciprocal relationship with cancer: the lower incidence of cancer among patients with Huntington disease

Normal HTT protein play significant role in transporting materials, chemical signaling, structural attachments and in preventing apoptosis. It also impacts on functioning of several transcription factors and many genes. Cortical neurons produce BDNF transcription, it enhances neuronal survival. Huntingtin is involved in this upregulation of BDNF transcription. When HTT gets mutated in Huntington disease, this activity of HTT also disappears and production of BDNF decreases. It has already been reported that there is decreased incidence of cancer in Huntington disease patients and this seems to be related to intrinsic biologic factors [3]. In Huntington disease, HTT protein is modified and it increases the rate of apoptosis, also provides protection against cancer. But this increased rated of apoptosis in Huntington disease causes neurodegeneration. It is important to note that BDNF, P53, increased apoptosis, transcription changes, are also among the aspects in which cancer and Alzheimer’s disease have reciprocal relationship.

### Weakened anti-stress responses such as vimentin, carnosine, stress protein expression, HSPs and ubiquitin

Anti-stress responses gets weakened with age, this in turn also contributes to the development of Alzheimer’s disease such as Vimentin has decreased expression in Alzheimer’s disease but over-expressed in Cancer, Carbonic anhydrases are increased in Cancers while decreased in AD brains. HSPs are increased in cancers but decreased in AD, SAPK downregulation occurs in cancer for survival of cancerous cells and SAPK inhibitors can prevent neuronal apoptosis in AD. All of these weakened anti-stress responses also signify the inverse relationship between Alzheimer’s disease and cancer. Carnosine that is an anti-aging homeostatic agent, neuro-protective and antineoplastic, its response also weakens with age, thus contributing to the development of AD. Carnosine and FOXO have complex relationship to both these diseases.

#### Vimentin has decreased expression in Alzheimer’s disease but over-expressed in cancer

In brain tissues from several human CNS and non-neurological disease cases, immunoreactivity of vimentin was highly positive in subpial and ependymal cells, but in some white matter astrocytes and in some capillaries, it was weakly positive. Vimentin- immunoreactivity in astrocytes, macrophages and microglia becomes intensely positive in areas of brain affected by Alzheimer’s disease, multiple sclerosis, Pick’s disease, amyotrophic lateral sclerosis and cerebral infarction patients. But in AD, they are found specially associated with beta amyloid plaques [71]. Before there is loss of neurons in AD, there is loss of synapsis, retraction of dendrites. Response of neurons to this development of AD pathology is still not properly known. In brains affected by AD, there is localization of vimentin to dendrites and perikariya of neurons. There commonly occurs co-localization of vimentin and beta amyloid plaques in hippocampus, cerebral cortex and in cerebellum. Even AD affected regions of transgenic mice, when mechanically injured, these regions expressed vimentin. During human embryonic development, vimentin expression occurs concurrently in relation to rapid extension of neurite. Expression of vimentin by neurons is an evolutionarily conserved mechanism to counter damage response which repeats a developmental pathway that differentiating neurons use in establishing connections in synapses and dendrites [72].

Vimentin provides protection against cellular stress and is also involved in maintenance of cellular integrity. It is important to note that dendrites and synaptic connections are among the most affected ones in AD. Growth of dendrites contributes to the learning and formation of memories. In AD, vimentin expression that has also been termed damage response mechanism is limited to Astrocytes, Microglia, and Beta Amyloid plaques, and also is not that much as in Ischemic brain Injury. Vimentin possibly also controls other proteins, it regulates transcription. It has multiple functions and is able to interact with several proteins, and potentially regulates several physiological functions. It contributes to maintenance of structural integrity of cells. Several other functions of vimentin are yet to be investigated [73].

This may point to new insights about AD. It perhaps is due to decreased expression of factors that are required to maintain neuronal homeostasis, such as decreased expression of vimentin that has damage response mechanisms that Alzheimer’s disease develops. As a person ages, neurons have to counter stress daily and they have mechanisms to counter this stress. The expression of such anti-stress mechanisms continue to decline with ongoing age and when such ongoing decline in expression reaches a certain point, neuronal damage begins to manifest clinically and AD signs and symptoms begin to appear. Expression of Vimentin is present in normal mesenchymal cells, neuronal precursor cells, sertoli cells, and fibroblasts. This protein is present both outside and inside the cells. Vimentin is an Intermediate filament (IF) protein and IF proteins play role in bringing about RNA and DNA-mediated events inside the cell nucleus. There are various cancers in which over-expression of vimentin is present including breast cancer, prostate cancer, lung cancer, CNS tumors, gastrointestinal tumors, malignant melanoma and other cancer types. This over-expression co-relates with upregulated growth of tumors and invasion [74].

#### Carbonic anhydrases are increased in cancers while decreased in AD brains

In AD brains, activity of carbonic anhydrase (CA) is decreases, and when carbonic anhydrase activators are administered, this contributes to improvement in learning in animals [75]. CA is activated by carnosine. Carbonic anhydrases are increased in Cancer [76].

#### Carnosine: an antiaging homeostatic agent that is neuro-protective and anti-neoplastic

Functioning and levels of carnosine deteriorates with age, and this contributes to AD and cancer development. Carnosine is neuroprotective, and also anti-proliferative. As both diseases are considered age-related, it is also anti- aging. And it has been found to be pro-apoptosis and anti–apoptosis as well. Beta amyloid toxicity can be suppressed by carnosine; a homeostatic, pluripotent, naturally occurring agent. It also scavenges reactive aldehydes and hydroxyl radicals, suppresses protein glycation, and inhibits the production of ROS. High levels of both zinc and carnosine are present in olfactory lobe. In AD, one of the early symptoms is the decline in olfactory function, and olfactory tissues are damaged by oxidative stress. Aging causes decline in the levels of homocarnosine in human CSF. In NFTs of AD brains, there is presence of gamma-glutamyl-epsilon-amino; protein cross-links. From biological tissue, gamma-glutamyl-carnosine has already been isolated. It is important to note that in cultured human fibroblasts, vimentin expression is stimulated by carnosine. There is also co-expression of vimentin with protease oxidized-protein-hydrolase. Carnosine is also involved in stimulating proteolysis in senescent cultured fibroblasts and in cultured myocytes [75]. Further exploration of carnosine and other such agents is needed, as they may potentially play role in development of new therapies towards neurodegenerative disorders including AD. Carnosine decreases growth of tumors and lowers the quantity of mitotic cells in them; this has been shown in animals. Carnosine negatively impacts the proliferation. It has high potential to halt the growth of malignant cells [77].

Carnosine has other significant beneficial effects too, such as prevents dysfunction of testicles caused by gamma-irradiation. It does this prevention because it has anti-apoptotic effect and it also lowers ROS. Carnosine also maintains normal levels of glutathione when the brain has suffered ischemia. It also provides neuroprotection [78].

#### Role of HSPs and inverse relationship between AD and cancer

Carnosine upregulates the gene expression of stress protein and synthesis of nitric oxide contributes to eliminate altered proteins proteasomally [79]. Synthesis of ‘stress proteins’ becomes elevated whenever cell is in conditions of stress. Stress proteins are also called heat shock proteins (HSPs), they have significant role in normal and injured cells. HSP expression increases in response to stress vectors including ischemia and high temperature [80]. This increase in gene expression of HSP is induced by heat shock factor (HSF). HSF 1 increases and maintains the gene expression of Hsp70. It is already known that specific neuronal structures such as synapses and axons also harbor HSPs that may be misregulated during the Alzheimer’s disease processes. HSF 1 has the powerful capacity to modify carcinogenesis. HSF1 is involved in promoting metastasis and survival in cancer cells. Inhibition of HSF1 impairs mitogenic (MAPK) signaling and contributes to inducing apoptosis in cancer cells [81]. It may also be possible that some of the HSPs may be upregulated to counter Alzheimer’s related damage, and this needs to be further investigated.

#### Ubiquitin levels are decreased in Alzheimer’s disease and increased in cancer, also signify the inverse relationship between Alzheimer’s disease and cancer

Ubiquitin is involved in marking protein for degradation. It also has properties of heat shock proteins (HSPs). In cancer cells, there is high expression of HSPs, and it contributes significantly to their survival. Gene expression is also regulated by ubiquitination of histones. Ubiquitination is also significantly involved in regulating transcription factors p53 and Myc [82]. When amyloid precursor protein (APP) becomes malformed, it contributes to AD development, but this malformation is decreased when higher levels of ubiquitin are present in the brains and lower levels increase the malformation of APP [83]. Ubiquitin also contribute to neuroprotection against ischemic injuries [84].

It’s important to note that carnosine upregulates stress proteins, and ubiquitin has also effects of stress proteins and they can modulate transcription and transcription factors including Myc and p53. Process of transcription is modulated by all these effects. Increased levels of ubiquitin have neuroprotective affects.

#### SAPK downregulation occurs in cancer for survival of cancerous cells and SAPK upregulation occurs in AD, also signifying the inverse relationship between cancer and Alzheimer’s disease

In AD, neuronal apoptosis is mediated by oxidative stress induced by beta amyloid plaques. Stress-activated protein kinases (SAPK) regulate mitochondrial pathways including Bcl2 and p53, which paly roles in apoptosis. SAPK upregulates p53 levels, but MAPK, p38 and JNKs are capable of specifically inhibiting SAPK and apoptosis [31]. When cells become cancerous, stress signaling is induced and levels of SAPKs and ROS are increased to contribute to apoptosis. But when tumors become advanced, this death signaling is down-regulated [85].

#### Normal functioning of cell mechanisms deteriorates with increasing old age, such as decreased regulation of signal transduction and gene expression occurs in AD brains

Vitamin E is a redox sensor and regulates gene expression and signal transduction. It has also been shown to decrease and slow the cognitive decline in AD patients when given in high doses [86].

#### FOXO helps in cell survival and p53 is involved in cell death; their relation to AD

Aging weakens the functioning of FOXO and thus this contributes to AD development. Ultimately this favors p53 upregulation, resulting in increased neuronal death and further acceleration of Alzheimer’s disease. Aging increases the risk of AD. FOXO is involved in countering stress, maintenance of neural stem cells and also contributes to longevity. It also play important role in cellular proliferation, metabolism and tolerance of stress. Ubiquitination, phosphorylation and acetylation and other post-translational modifications are involved in tightly regulating the activity of FOXO. Many enzymes that contribute to post-translational modifications are shared between p53 and FOXO, and they affect both in opposing manner. This may underlie a balance between lifespan and disease [87].

### The following measures are possibly the ‘Countermeasures or compensatory mechanisms by AD affected neurons’ as last attempts for survival which may be protective for certain time such as Tau, Beta Amyloid, S100 – or can speed up AD in the Alzheimer’s disease microenvironment via C-ABL activation, GSK3, neuro-inflammation

Cellular pathways involved in growth, proliferation, development, gene expression and survival, are utilized as compensatory mechanisms by AD neurons, and this helps in survival for a limited time as the degenerating environment becomes more toxic, then they ultimately contribute to further AD progression. Some of the key pathways that may be playing compensatory roles have been pointed in this study. Roles of all these pathways as compensatory mechanisms or countermeasures have been proposed in the light of scientific literature. And all these roles need to be further investigated also via using AD animal models.

#### GSK3 involved in neurodevelopment and it also appears in Alzheimer’s disease, but in AD the microenvironment is neurotoxic so ultimately it speeds up AD

GSK3 is involved in pathogenesis of AD. GSK3b involved in proliferation, differentiation, motility and survival during embryogenesis. Mdm2 is responsible for degrading p53 proteasomally, in physiologic conditions. But in times of cellular stress, there is stabilization of p53 and a complex formation with GSK3β. This causes hyper-phosphorylation of tau, enhanced accumulation of beta amyloid plaques and upregulation of GSK3β. P53 is upregulated and its proteasomal degradation is inhibited in the presence of beta amyloid plaques. It is also considered that phosphorylated p53 and GSK3β work together in the development of AD. NFTS and beta amyloid plaques are considered among the principal components of AD. It is assumed that NFTs and plaques may be responsible for neuro-toxicity or they may actually be a mechanism to provide neuro-protection [5]. Glycogen synthase kinase 3 (GSK3) is involved in neuro-developmental fundamental processes, and if this signaling is disrupted, it then contributes to cause neurodevelopmental disorders. GSK3 signaling contributes to development of neurons, their polarization and growth of axons, when brain is in the phase of development. It is involved in mediating multiple cellular processes and impacts several signaling molecules [88, 89].

Physiologically, GSK3 contributes to maintenance of cell structure, gene expression, apoptosis and metabolism. GSK3 signaling is extremely important in neural development and perhaps in order to repair the damage and to protect the cells from AD neuro-degeneration, this protein appears in AD brains but micro-environment is now quite different as compared to the time of embryogenesis, and in AD p53 gets stabilized forming a complex with GSK3β and contributes to neuronal death, ultimately speeding up AD.

#### C-Abl is involved in neurodevelopment and is turned up in CML but it is also present in AD brains, not halting AD

C-Abl protein is a tyrosine kinase. It plays significant roles in developmental pathways including cellular proliferation, adhesion and differentiation. C-Abl turns on the cell cycle in the brain of adults, this damages the cells and cause phosphorylation of tau [90]. C-Abl is also present in beta amyloid plaques and NFTs in AD brains. It is significant to point out that this protein is upregulated in B cells in chronic myeloid leukemia (CML). It is hypothesized here that as Alzheimer’s disease halts neurogenesis, so alternatively C-Abl here is utilized to serve the purpose turn on the cell cycle but in adult brain it damages the cells and further accelerates the AD, as the cell environment and surrounding microenvironment is neurotoxic.

#### Neuro-inflammation, S100B, Down syndrome and Alzheimer’s disease

Down syndrome halts growth and also causes early Alzheimer’s disease. Among defects Trisomy 21 causes premature aging and mental retardation. It is also considered possible that initial events in AD pathogenesis are driven by cytokines, because there occurs inflammation of neurons, glial cells dramatically multiply and over-express IL-1 and S100B in the brains of down syndrome patients [91]. Interleukin (IL)-6, a marker for neuro-inflammation, is found in brains of AD and DS. IL-6 is involved in modulation of growth and differentiation in various malignancies. Estrogen, whose role has previously been mentioned as anti- AD, is inhibitor of IL6.

Physiologically, S100B regulates proliferation of cells and axons, differentiation, neurite extension, astrocytosis and other cellular processes. It also functions as a survival signal and neurotrophic factor. It is important to note that S100B and its receptor is upregulated in AD [92].

Down syndrome cause early onset AD, both the Trisomy 21 and AD have defective growth, proliferation, maintenance and survival responses in many aspects. It may be hypothesized that as patient’s brain cells need mechanisms for proper regulation of growth, survival and maintenance of brain cells, but Trisomy 21 tends to halt these responses. In order to compensate this effect of Trisomy 21; IL- 1, IL -6 and S100B are utilized to serve this purpose. IL – 1 and IL - 6 also results in neuro-inflammation and accumulation of plaques in neurotoxic micro-environment of AD, ultimately speeding up AD.

S100B is utilized for neural growth and maintenance but is insufficient to stop progression of AD damage.

#### SPs, NFTs and GVDs are also found in normal aging brains

Growth, proliferation, survival and maintenance mechanisms are impaired in AD brains, it is perhaps because of this reason that growth of dendrites is also impaired compared to healthy individuals. SPs and NFTs both have been found in healthy brains. They also have roles in developmental processes, gene expression and survival. Kinases are known to play roles in cell signaling and carcinogenesis, and Kinases such as MARK 3-4 have also been found in GVDs. The presence of SPs, NFTs and GVDs in AD brains may actually be a compensatory response to aid in preventing AD related damage to brain structures but as the micro-environment becomes neurotoxic, they ultimately speed up AD.

Tau Changes in healthy persons are involved in regulating developmental processes and gene expression [93]. Many studies have already suggested that phosphorylation of tau is significant for generation of neurons in hippocampal region [94]; some neurons containing NFTs survive for decades and a great number of neurons are already lost before NFTs appear. There is also possibility that NFTs are not directly related to degeneration of neurons as they have already been observed in seemingly healthy people. Therefore, it has already been suspected and proposed that NFTs form as a compensatory mechanism after oxidative damage, and this damage results in activation of multiple kinases which led to tau phosphorylation. This formation of NFTs prolongs the normal functioning of neurons for a certain period of time. The role of micro-environment in normal functioning, development and maintenance of tissues is very significant. Beta amyloid plaques may impact AD- associated genes transcriptionally. Beta-Amyloid also may be part of mechanism to counter aging related neuronal troubles, as SPs are also found in normal aging brain. GSK3beta contributes to development of AD and its role has already been mentioned. It interacts with beta amyloid plaques, NFTs, presenilin and with other AD related proteins. APOE4 increases the risk of AD, only the risk not the AD itself [5].

Granulovascular Degeneration Bodies (GVDS) are rarely seen before age 65, but become increasingly common with age. GVDs contain increased MARK 4 and then MARK 3 in AD patients compared to non-demented elderly. Microtubule affinity regulating kinase (MARK) phosphorylates Ser^262^ site on tau, this phosphorylation is correlated with the expressions of MARK3 and MARK4 in GVDs in AD brains, and detaches tau from microtubules. This detached tau then becomes available for more phosphorylation [95]. Drug which modulated gamma secretase and also blocked complex of PS1 to avoid production of beta amyloid plaques, failed fully to halt AD [96]. If beta amyloid plaques had been the key culprit behind AD, then preventing Aβ 1–42 production would have already been successful in preventing cognitive decline in AD patients.

It has already been discovered on the basis of post mortem studies, beta amyloid plaques may appear in healthy elderly brains and may be absent even in highly progressed AD cases. Hallmarks of Ad are also in76% of normal persons.

Physiologic functions of Aβ include kinase activation, ion channel formation, cholesterol transport regulation and neuro-protection against oxidative stress to some extent. APP and Tau are involved in brain development, gene expression but when are impaired, they contribute to Alzheimer’s disease progression. APP plays significant roles in proper neuronal functioning and in brain developmental pathways, but in AD it changes to βAP. And this change into βAP may be a compensatory response to counter AD-related damage.

#### Tau a microtubule associated protein: role, and its relation to Alzheimer’s disease and cancer

Hyperphosphorylated tau as NFTs accumulates in cerebral cortex and hippocampal regions of the brain in AD patients. When tau becomes hyperphosphorylated, it then may become unable to bind properly to microtubules and ultimately resulting in their collapse. Roles of microtubules are well known in developmental processes and in regulation of gene expression. Microtubules and associated proteins such as MAPs play very significant role in the development of CNS. They and tau are present in high quantities during the development of brain and are also involved in crosslinking microtubules inside the neurons. Tau works in relation to tubulin in cross-liking process [97]. AD is associated with accumulation of beta amyloid plaques and tau hyperphosphorylation. Generation of new neurons becomes impaired in AD. It is important to point out here that phosphorylation of tau significantly contribute to neurogenesis in hippocampal region of brain [98]. Cytoskeleton of cells including microtubules regulate expression of genes, support intracellular structures and transport, contribute to signal transduction and to generation of some growth factors including connective tissue growth factor.

It is significant to note that antineoplastic drugs acting on microtubules, harm the cell survival and proliferation and ultimately led to cell death of cancer cells [99–101]. This signifies the growth maintaining and promoting role of microtubules and tau. With increasing age, this role also deteriorates and increased hyper-phosphorylation of tau takes place. Tau protein is involved in developmental processes, differential gene expression and growth factors production, and tau phosphorylation is also involved in neurogenesis. Its accumulation as NFTs in AD brains may actually be a compensatory response by neurons to prolong their survival in AD brains, but ultimately the degenerating environment makes this response insufficient and contributes to AD progression. Many processes that are involved in growth, development, and neoplasia are down-regulated or deteriorate in AD, and pointing this out is one of the aims of this study.

## Discussion

This research article attempts to investigate what causes and makes the Alzheimer’s disease process begin. Understanding the relationship between cancer and AD, in terms of cellular pathways and molecular mechanisms will help to understand better the pathogenesis and factors behind Alzheimer’s disease. This study also investigates how age related changes in growth, cell survival responses, maintenance mechanisms, anti-stress responses, contribute to development and progression of Alzheimer’s disease.

In the light of scientific literature, this research paper also points to possible mechanisms and pathways that may be playing role or contributing to Alzheimer’s disease pathogenesis. But these possible mechanisms and pathways need to be further investigated. Cancer and Alzheimer’s disease have inverse relationship and all the factors that are inversely related, also other factors and changes that lead or contribute to the development of AD may also be new potential tools for diagnostic testing especially for Alzheimer’s disease and also in designing new potential interventions and treatments for both diseases but especially for Alzheimer’s disease. Understanding both these age-related diseases may also provide deep insights into the biology of aging. This study may also potentially provide new insights into biology of aging. This research article may help answering the questions. Such as, why does AD affect some aged people but not everyone? How genes contributing to AD interact with environmental and lifestyle factors and other genes, and how this impacts the risk of AD development? Who are more likely to develop AD?

All those factors that contribute to growth and proliferation are increased in Cancer but decreased in Alzheimer’s disease. This simply does not mean that every cellular or molecular pathway should have inverse relationship; there are so many pathways that are common and even operate similarly in many cell types, and are not altered by disease processes. Some of the key pathways that may be playing compensatory roles have been pointed in this study. It is important to note that cancer is not a single disease; there are multiple types of cancers. Roles of all the pathways as compensatory mechanisms or countermeasures have been proposed in the light of scientific literature. And all these roles need to be further investigated also via using AD animal models. It also suggests some hypothesis in the light of scientific literature and also highlights the changes occurring at cellular and molecular level esp. in AD.

This research study has taken a different approach and aims to further advance the understanding of AD development and progression. This paper cites evidence in support of this study from already well-established and published research literature. Further research and experimental studies at molecular, genetic, epigenetic and cellular levels, will reveal deeper details about the findings presented in this study. This may take our understanding of Alzheimer’s disease, Cancer and Aging biology to greatest depths. Experiments should also be devised so as to investigate which factors greatly contribute to AD or are causative directly or indirectly. Investigating aging in greater depths will also lead to better understanding of age-related diseases such as Alzheimer’s disease.

## Conclusions

It’s the age-related cellular and genomic changes that when becomes pathologic to such an extent that age-related diseases such as Alzheimer’s disease and cancer appear. When mechanisms involved in physiologic growth, development, maintenance, proper gene expression are impaired, then they result in AD. Aging greatly contributes to these impairments. Alzheimer’s disease and cancer, both are age related diseases, one is degenerative and other is over-proliferative. Many factors that are upregulated in any Cancer to sustain growth and survival are downregulated in Alzheimer’s disease contributing to neuro-degeneration.

There is inverse relationship between Cancer and Alzheimer’s disease in aspects such as P53, estrogen, neurotrophins and growth factors, growth and proliferation, cAMP, EGFR, Bcl-2, apoptosis pathways, IGF-1, HSV, TDP-43, Alzheimer’s risk decreases from apoE4 to E3 to E2 but growth and survival improves respectively, notch signals and presenilins, NCAM, TNF alpha, PI3K/AKT/MTOR pathway, telomerase, ROS, ACE levels.

Anti-stress responses gets weakened with age, this in turn also contributes to the development of Alzheimer’s disease such as Vimentin, Carbonic anhydrases, HSPs, SAPK. All of these weakened anti-stress responses also signify the inverse relationship between Alzheimer’s disease and cancer.

Carnosine that is an anti-aging homeostatic agent, neuro-protective and antineoplastic, its response also weakens with age, thus contributing to the development of AD. Carnosine and FOXO have complex relationship to both these diseases.

There are ‘Countermeasures or compensatory mechanisms by AD affected neurons’ as last attempts for survival which may be protective for certain time such as Tau, Beta Amyloid, S100 or can speed up AD in the Alzheimer’s disease microenvironment via C-ABL activation, GSK3, neuro-inflammation.

Cellular pathways involved in growth, proliferation, development, gene expression and survival, are utilized as compensatory mechanisms by AD neurons, and this helps in survival for a limited time as the degenerating environment becomes more toxic, then they ultimately contribute to further AD progression.

This study is important as it signifies inverse relationship between AD and cancer via elaborating cellular and molecular pathways that are responsible for this relationship. It also points to other significant factors and their roles in AD development and progression. It signifies how deteriorating growth and survival mechanisms contribute to AD, providing new insights into AD pathogenesis.

## Study design

The etiology of Alzheimer’s disease is yet not properly known. This research study finds evidence from already published research literature to find factors and changes that lead to the development of Alzheimer’s disease and finds the inverse relationship between Cancer and Alzheimer’s disease.

It investigates the cellular pathways having roles in cancers, development, aging, cell survival, growth and proliferation, and also contributing to Alzheimer’s disease (Additional file 1).

## Additional file

Additional file 1: PRISMA Flow Diagram. (JPG 139 kb)

## Abbreviations

Abeta: Beta amyloid
ACE: Angiotensin-converting enzyme
AD: Alzheimer’s disease
APOE: Apolipoprotein E
APP: Amyloid precursor protein
Aβ42: Amyloid beta 1–42
BACE: Beta-secretase
Bcl-2: B-cell lymphoma 2
BDNF: Brain-derived neurotrophic factor
BMP: Bone morphogenetic protein
CA: Carbonic anhydrase
cAMP: Cyclic adenosine monophosphate
Cdk 6: Cyclin-dependent kinase
COX: Cyclooxygenase
DGCs: Dentate granule cells
EGF: Epidermal growth factor
FOXO: Forkhead box O
GSK: Glycogen synthase kinase
GV: Granulovascular
GVDs: Granulovascular degeneration bodies
HSPs: Heat shock proteins
HSPs: Heat shock proteins
HSV: Herpes simplex virus
IGF: Insulin-like growth factor
IL: Interleukin
JNK: c-Jun N-terminal kinases
LTP: Long-term potentiation
MAPK: Mitogen-activated protein kinase
MAPK: Mitogen-activated protein kinases
MAPs: Microtubule-associated proteins
MARK: Microtubule affinity regulating kinase
mTOR: Mammalian target of rapamycin
NAC: N-ACETYLCYSTEINE
NCAM: Neural cell adhesion molecule
NFT: Neurofibrillary tangles
NF-κB: Nuclear factor-kappa B
NGF: Nerve growth factor
NGF: Neural growth factor
NSCs: Neural stem cells
NT: Neurotrophins
*OXPHOS*: *Oxidative phosphorylation*
PC: Pheochromocytoma
PI3K: Phosphoinositide 3-kinase
PKC: Protein kinase C
PS1: Presenilin 1
ROS: Reactive oxygen species
SAPK: Stress-activated protein kinases
Shh: Sonic hedgehog
SPs: Senile plaques
TBI: Traumatic brain injury
TDP 43: TAR DNA-binding protein 43
TNF: Tumor necrosis factor
TUNEL: Terminal deoxynucleotidyl transferase dUTP nick end labeling
XIAP: X-linked inhibitor of apoptosis protein
