# Supplementary figures and images for: Inverse relationship between Alzheimer’s disease and cancer, and other factors contributing to Alzheimer’s disease: a systematic review
Source: BMC Neurol. 2016 Nov 22;16:236. doi: 10.1186/s12883-016-0765-2 (PMC5120447; doi:10.1186/s12883-016-0765-2)

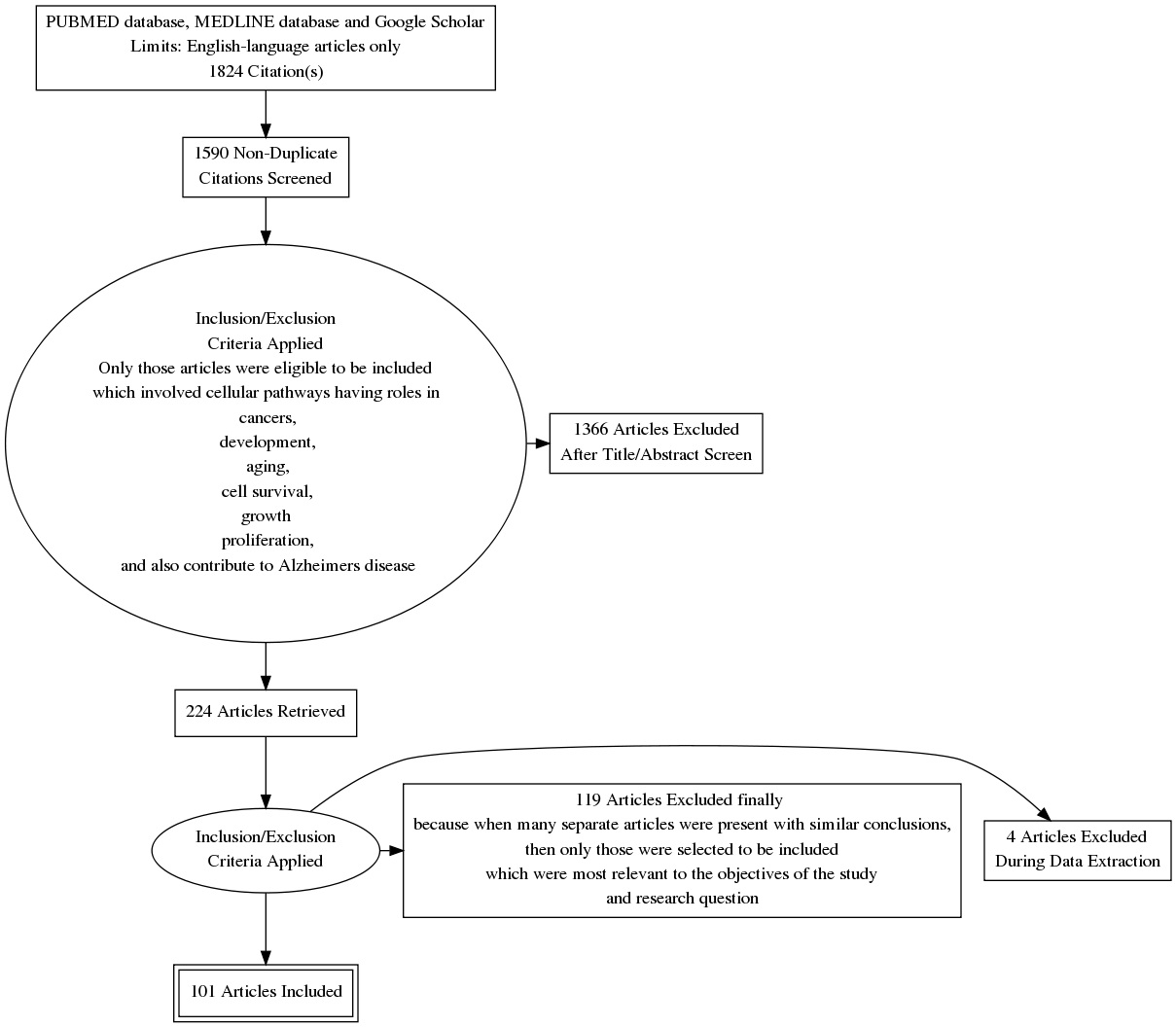

Supplement: Additional file 1: — PRISMA Flow Diagram. (JPG 139 kb) [file 12883_2016_765_MOESM1_ESM.jpg]
